# Bone Disease in Primary Hyperparathyroidism—Changes Occurring in Bone Metabolism and New Potential Treatment Strategies

**DOI:** 10.3390/ijms252111639

**Published:** 2024-10-30

**Authors:** Mirella Iwanowska, Magdalena Kochman, Alicja Szatko, Wojciech Zgliczyński, Piotr Glinicki

**Affiliations:** 1 Department of Endocrinology, Centre of Postgraduate Medical Education, 01-813 Warsaw, Poland; 2 EndoLab Laboratory, Centre of Postgraduate Medical Education, 01-809 Warsaw, Poland

**Keywords:** primary hyperparathyroidism, osteoporosis, parathyroid hormone, PTH, bone metabolism

## Abstract

Primary hyperparathyroidism (PHPT) is a common endocrinopathy, predominantly caused by a single parathyroid adenoma that is responsible for the excessive secretion of parathyroid hormone (PTH)—the hallmark of disease. Excess of this hormone causes remarkable changes in bone metabolism, including an increased level of bone remodeling with a predominance of bone resorption. Those changes lead to deterioration of bone structure and density, especially in cortical bone. The main treatment for PHPT is surgical removal of the adenoma, which normalizes PTH levels and terminates the progression of bone disease and leads to its regeneration. However, because not all the patients are suitable candidates for surgery, alternative therapies are needed. Current non-surgical treatments targeting bone disease secondary to PHPT include bisphosphonates and denosumab. Those antiresorptives prevent further bone loss, but they lack the ability to regenerate already degraded bone. There is ongoing research to find targeted drugs capable of halting resorption alongside stimulating bone formation. This review presents the advancements in understanding the molecular mechanisms responsible for bone disease in PHPT and assesses the efficacy of new potential therapeutic approaches (e.g., allosteric inhibitors of the PTH receptor, V-ATPase, or cathepsin inhibitors) aimed at mitigating bone loss and enhancing bone regeneration in affected patients.

## 1. Introduction

Primary hyperparathyroidism (PHPT) is one of the most common endocrinopathies. The prevalence of this disease largely depends on the geographic region: it is highest in North America and Western Europe (with approximately 233 per 100,000 in women and 85 per 100,000 in men in the USA). In contrast, the prevalence of PHPT in developing countries is much lower, primarily due to the lack of routine biochemical diagnostics [1].

The most common cause of PHPT is a single parathyroid adenoma [2]. Parathyroid adenoma develops primarily due to excessive, uncontrolled proliferation of parathyroid cells and changes in the expression of genes responsible for producing calcium-sensing receptors (CaSR) on their surface. The quantity and activity of these receptors decrease, and as a result, there is a pathological enlargement of the parathyroid gland, decreased cellular sensitivity to hypercalcemia, and excessive secretion of parathyroid hormone (PTH) [1]. Alongside the reduction in CaSR, the number of vitamin D receptors (VDRs) in parathyroid adenoma is also decreased. This leads to a failure in their stimulation by calcitriol, exacerbating the down-regulation of genes responsible for CaSR production and negatively impacting PTH inhibition [3]. Other less common causes of PHPT include parathyroid hyperplasia, often observed in multiple endocrine neoplasias types 1 and 2a (MEN1, MEN2A), and inactivating heterozygous mutations of the CaSR gene, associated with most cases of familial hypocalciuric hypercalcemia (FHH).

Under physiological conditions, PTH primarily ensures the maintenance of normocalcemia and affects bone remodeling processes, influencing both bone formation and resorption. Excessive PTH-mediated stimulation leads to varying degrees of hypercalcemia, primarily associated with increased bone resorption and enhanced renal calcium reabsorption. The development of hypercalcemia is intensified by elevated calcitriol levels due to increased 1-alpha-hydroxylase and decreased 24-hydroxylase activity, both triggered by heightened PTH stimulation in the kidneys. High levels of calcitriol observed in PHPT patients are associated with increased markers of bone remodeling and negatively impact bone mineral density, particularly in the forearm and hip regions [4,5]. Elevated serum calcium levels can lead to the development of hypercalcemic crisis, nephrocalcinosis, and kidney stones, while changes in bone metabolism can result in decreased bone mineral density, radiological features of osteitis fibrosa cystica [6], and an increased risk of fractures [7,8].

Changes in bone metabolism in the course of PHPT are associated with the continuous effect of PTH stimulation on bone tissue, leading to increased bone turnover and a predominance of bone resorption over formation. Resorptive processes are so intense that, even though the so-called “coupling” phenomenon occurs—where bone formation processes are also induced in areas of increased bone resorption—their intensity is insufficient relative to the catabolic processes [9]. Continuous PTH (cPTH) stimulation on cells present in bone tissue (osteoblasts, osteocytes, osteoclasts, skeletal stem cells, etc.) activates molecular pathways responsible for the characteristic changes in bone observed in PHPT. This is typically reflected by higher serum levels of bone turnover markers (e.g., C-terminal telopeptide, procollagen type 1 N-terminal propeptide, alkaline phosphatase) [10,11], which also serve as indicators of the severity of bone tissue disturbances [11]. Those bone effects of cPTH can be observed in PHPT caused by parathyroid adenoma as well as in parathyroid hyperplasia (typical for MEN syndromes) [12]. In MEN1 syndrome, patients can experience more severe bone demineralization, probably as a consequence of prolonged exposure to cPTH resulting from the earlier onset of the disease compared to the sporadic form of PHPT [13]. In MEN2A, the course of PHPT is usually milder than in MEN1 [14]. On the other hand, despite elevated PTH levels in FHH, bone structure does not appear to be impaired, and an increased incidence of fractures is not observed. This may be related to the important role of CaSR, which is expressed on both osteoclasts and osteoblasts and can influence bone remodeling [15,16].

The treatment of choice that offers the possibility of a complete cure remains surgical intervention [17], specifically the removal of a single parathyroid adenoma in most cases. However, the safe conduct of a parathyroidectomy (PTX), like any other surgery, requires proper patient’s preparation for general anesthesia, primarily through the management of chronic comorbidities. A thorough assessment of the benefits and the risks associated with surgical treatment is essential. This includes estimating the potential for full recovery after surgery, analyzing the likelihood of adverse events occurrence, and determining the possibility of preventing them. Many patients with PHPT have a mild course of the disease, and the complications are limited to bone loss. Those patients are usually elderly, with multiple comorbidities, and sometimes have undergone previous neck surgery. Due to their accompanying illnesses and increased perioperative risk, they are more susceptible to dangerous complications related to anesthesia and PTX, which may result in the benefits of such treatment not outweighing the potential side effects. Consequently, the latest guidelines for managing patients with PHPT [17] allow for symptomatic treatment of osteoporosis secondary to it using bisphosphonates or denosumab. Pharmacological treatment is an important alternative to routine surgical management for the patients who are not candidates for surgery. Since those patients represent a significant part of individuals with PHPT, and due to the known limitations of the antiresorptive drugs currently used in this therapy, alternative treatment methods are being sought. Optimally, novel therapies should aim not only to stop the loss of bone density secondary to excess levels of PTH but also promote the regeneration of lost bone mass. At the same time, targeted therapies should have minimal side effects and interactions with other medications.

In this review, we aim to present the changes in bone metabolism that occur in the course of PHPT and prospects for the development of new therapies that may be applied in PHPT bone disease. This review begins with a presentation of the general bone changes observed in PHPT. Secondly, we focus on the molecular mechanisms involved in increased osteoclastogenesis and heightened osteoclast resorptive activity in PHPT. Finally, the effects of cPTH on bone formation processes are discussed. At each part, we highlight the potential therapeutic approaches relevant to the molecular mechanisms described. The therapies described in the paper, along with their mechanisms of action, are summarized in the table below (Table 1).

## 2. Bone Disease in PHPT

In the course of PHPT, bone loss particularly affects the cortical part of the bone [18]. It is often reflected by a remarkably reduced bone mineral density (BMD) in the forearm, as observed in densitometric studies (DXA) compared to the relatively preserved BMD of the bone in the spine, where cancellous bone predominates. In the region of the femoral neck, which is composed of both cortical and cancellous bone, the changes in BMD are intermediate between the previously mentioned areas [18]. Additionally, studies evaluating bone microstructure (histomorphometry of bone biopsy samples) have shown a depletion in bone thickness and increased porosity in cortical bone [18]. Conversely, the volume of cancellous bone and its microarchitecture could be even greater than in the group without PHPT. This phenomenon may be explained by increased formation bone turnover dynamic parameters in trabecular bone [18]. In clinical practice, however, such typical bone changes are not always observed. Depending on the clinical situation and the presence of other comorbidities and risk factors, such as low postmenopausal estrogen levels, years before PHPT diagnosis or advanced age, a decrease in BMD can also be observed in areas where cancellous bone predominates [17,19,20]. Mentioned co-morbidities can potentially mask the characteristic bone loss pattern of PHPT. Moreover, in severe and long-standing cases of PHPT, a markedly reduced BMD can be expected across all regions as excessive reduction in cortical and cancellous bone can occur [21]. Recent improvements in non-invasive bone imaging, such as trabecular bone score (TBS) and peripheral quantitative computed tomography (pQCT), have provided profound insights into bone quality and structural integrity in PHPT. TBS, which is a non-invasive method derived from lumbar spine DXA images that reflects trabecular microarchitecture and bone strength, is often degraded in PHPT despite normal spine BMD, indicating reduced bone quality. Alongside BMD, TBS could be a good predictor of vertebral fractures [22]. Using pQCT, which provides additional information about bone geometry and the quality of bone at peripheral sites such as the forearm or the tibia, it was assessed that PHPT patients have reduced both cortical and trabecular volumetric bone density (vBMD) and disturbed trabecular and cortical microarchitecture and geometry, especially in the radius [23]. When it comes to geometry and microarchitecture, PHPT patients had larger total bone areas and circumferences, indicating that excess PTH stimulates periosteal bone formation. However, cortical area and thickness were lower in PHPT patients, resulting in thinner cortical bone [23]. All above-mentioned changes in bone structure lead to increased risk of fractures in PHPT. Studies confirm higher total fracture risk with significant prevalence at the forearm and vertebrae [7,8].

## 3. Changes in Bone Metabolism Due to PHPT

### 3.1. Molecular Mechanism Involved in Increased Osteoclastogenesis and Osteoclast Differentiation in PHPT

Numerous studies have demonstrated that cPTH exposure in bone tissue leads to an increased level of bone remodeling [24,25,26,27], mainly due to increased osteoclastogenesis, and could be reflected by higher markers of bone turnover in blood and urine in comparison with controls [11]. Both anabolic markers (such as Alkaline Phosphatase, Bone-Specific Alkaline Phosphatase and Procollagen Type 1 N-Terminal Propeptide) and catabolic markers (such as C-terminal and N-terminal telopeptides of type I collagen) are elevated [11], albeit the catabolic effects on bone predominate. Excessive PTH levels in PHPT, by binding to its receptors (parathyroid hormone receptor 1—PTH1R), enhance osteoclastogenesis through the intensive activation of macrophage colony-stimulating factor (M-CSF) [28] and receptor activator of nuclear factor kappa-B ligand (RANKL) transcription and decreased osteoprotegerin (OPG) production by osteoblasts [24,25,29]. The presence of receptor activator of nuclear factor κB (RANK) and colony-stimulating factor receptor 1 (CSF1R) on pre-osteoclasts leads to increased proliferation, maturation, and activation of osteoclasts.

The increased number of activated osteoclasts amplifies the number of bone metabolic units (BMUs) and increases the area of bone resorption [30]. Although osteoblasts are also present and active in these new BMUs [15,26,27], their activity does not compensate for the intensive osteoclastic action. This imbalance results in a net increase in bone resorption over bone formation.

Currently available medications recommended in treatment of PTHP-mediated bone disease (when surgery is not eligible) [17] include bisphosphonates or denosumab. The above-mentioned medications act as antiresorptives by suppressing osteoclast differentiation and activity and decreasing their number. Bisphosphonates bind to the bone hydroxyapatite, then they are absorbed by mature osteoclasts during the process of bone resorption, and as a result, they inhibit the activity of osteoclasts and promote their apoptosis. Denosumab is a human monoclonal antibody that functions as a RANKL inhibitor. By blocking the interaction between RANKL and RANK on osteoclasts, denosumab effectively ceases the maturation, survival, and function of osteoclasts [31]. Both bisphosphonates and denosumab reduce bone turnover mainly by inhibiting bone resorption. They are capable of decreasing new BMU formation and further deterioration of already existing ones. As a result, they can preserve the BMD and microarchitecture of trabecular and cortical bone. Additionally, to some extent, osteoblasts present in BMUs are able to fill in the resorption pits. Although the bone formation abilities of those drugs are scarce, they cannot fully rebuild or regenerate already lost bone mass [32]. Furthermore, there are some considerations that completely inhibiting resorption processes for a long time causes bone inability to repair damage and, as a result, leads to bone weakening due to accumulation of injuries. Therefore, therapies are needed that, in addition to limiting resorption processes, can also have a positive effect on bone formation and maintain bone remodeling at an appropriate level.

Cinacalcet, which is a CaSR positive allosteric modulator, was found to be effective in decreasing elevated calcium levels in patients with PHPT by enhancing the CaSR sensitivity to extracellular calcium on the parathyroid gland and affecting the production of PTH. It can be used as a bridge therapy before PTX and sometimes as an alternative for those patients who cannot undergo surgery [17]. However, studies indicate that cinacalcet alone does not improve BMD in patients with PHPT [33,34], but in combination with antiresorptive agents it has been established as an effective strategy to control hypercalcemia and enhance bone density [35,36].

Avoiding vitamin D deficiency in PHPT patients was shown to reduce the severity of bone disease, as reflected by higher BMD values, and to lower PTH levels and bone turnover markers [37]. It is important to notice that during vitamin D supplementation, despite the baseline hypercalcemia, significant increases in calcium levels are not observed [38]. However, treatment with vitamin D is insufficient to completely prevent the progression of bone mass loss in the course of PHPT.

#### 3.1.1. Parathyroid Hormone 1 Receptor (PTH1R)

PTH is a peptide hormone that acts mainly through activation of PTH1R. This receptor is highly expressed in bones and kidneys, and its role is to maintain calcium homeostasis and regulate bone metabolism and skeletal development. Within bones and bone marrow, PTH1R is present on the surface of many bone cells, including skeletal stem cells, osteoblasts, osteoclasts, osteocytes, as well as adipocytes and endothelial cells [39,40,41,42]. The protein is encoded by the PTH1R gene, and it is a member of the G-protein coupled receptor family 2. The effect obtained after PTH binding to PTH1R depends on the duration of exposure to this hormone. When it is intermittent, then anabolic effects predominate. In contrast, when the exposure is continuous (like in PHPT), primarily catabolic actions are present, which leads to bone loss [43]. Studies evaluating the expression of the PTH1R gene revealed that cPTH exposure results in sustained high levels of PTH1R expression and increased receptor production [44,45].

Due to the presence of PTH1R on the surface of osteoclasts [39,40,42], they directly respond to cPTH stimulation and increase their resorption activity [39]. The effect of such stimulation could be increased expression of subunits of the V-ATPase, more intensive acidification inside the cells, and higher V-ATPase activity [46]. However, a key role in osteoclast proliferation and activation is influenced by their interactions with osteoblasts. PTH binding to PTH1R on osteoblasts leads to Gαs-mediated production of cAMP and then activation of protein kinase A (PKA), which is considered to be the major pathway of PTH signaling. Although PTH also stimulates other G-protein pathways like Gαq-mediated activation of phospholipase C and protein kinase C [47,48], the main pathway engaged in PTH-induced catabolic responses and osteoclastogenesis is through cAMP and PKA activation [29,44]. PKA phosphorylates many transcription factors, including cAMP response element binding protein (CREB), which promote expression of genes involved in bone resorption like RANKL. It is also involved in down-regulation of OPG production [29].

Inhibition of PTH1R may be a potential pharmacological target in treating illnesses associated with excessive receptor stimulation, like in PHPT. This effect was accomplished by certain agents that directly attach to the receptor to inhibit the binding and activation by agonists—such as competitive antagonists of PTH1R signaling [49].

Molecules that act by antagonizing the protein’s active site are called orthosteric antagonists. Among them, SW106 was found to have antagonist action; however, it had weak affinity and efficacy [50]. Another antagonist called DS08210767, which is a novel 1,4-benzodiazepin-2-one-based PTH1R antagonist, was found to have higher antagonist activity than the previous compounds and good oral bioavailability [51]. The success of DS08210767 led to the development of the optimized compound called DS37571084, which presented enhanced chemical stability, aqueous solubility, metabolic stability, and a superior pharmacokinetic profile [52]. Additionally, fully human, high-affinity anti-PTH1R antibodies developed by XOMA demonstrated blocking activation of PTH1R by PTH [53]. Although the ability of orthosteric modulators to reverse the effect of cPTH in PHPT patients needs to be further investigated in clinical practice, the direct deactivation of the PTH1R seems to be particularly useful in treating hypercalcemia associated with PHPT, especially in cases of life-threatening hypercalcemic crisis. These agents may effectively inhibit osteoclast activity (by directly blocking PTH binding to PTH1R on their surface and reducing V-ATPase activity), leading to a rapid reduction in serum calcium concentration. Additionally, by inhibiting PTH1R on osteoblasts, they could suppress RANKL production and reduce the differentiation of new osteoclasts. This antiresorptive action may also potentially enhance bone density parameters. However, such sustained inhibition of the influence of PTH on bone cells, particularly osteoblasts, could, in the long term, lead to a significant reduction in bone remodeling, insufficient new bone formation, and, as a consequence, to deterioration of bone quality.

Another potential way to affect PTH1R is to use the allosteric modulators to fine-tune the receptor’s activity. Allosteric modulators are the molecules that can bind to specified regions on a receptor (at a site other than the protein’s active site in contrast to orthosteric modulators) and modify the receptor’s conformation and activity. Positive modulators enhance, while negative modulators inhibit the binding affinity and catalytic activity of the receptor. The fact that they do not bind directly to the active sites of the receptor prevents its complete inhibition and could potentially reduce their side effects. In PTH1R, the extracellular ends of transmembrane regions TM1 and TM2 were found to be allosteric regions and can act as a drug target. The binding of a negative allosteric modulator (NAM) to PTH1R leads to conformational changes and reduced binding affinity between PTH and PTH1R. This results in decreased receptor activation and subsequently reduced G protein signaling [54]. Mengrong Li et al. described small molecule Pitt12, which binds mainly to the TM1 region. Acting as a NAM, it was shown to inhibit the PTH-induced cAMP signaling in cultured cells and to reduce the PTH effects on serum calcium and phosphate in mice [50].

**Figure 1:**
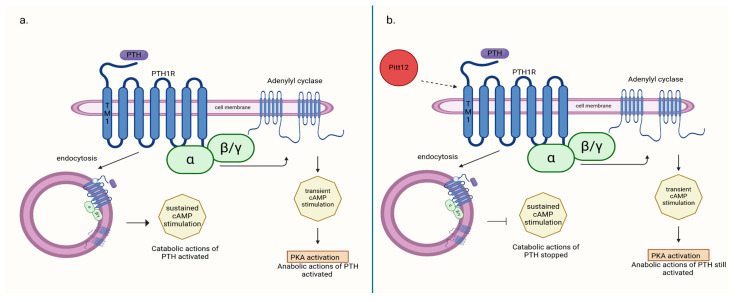
(**a**) PTH-induced catabolic action activated through cAMP production from sources generated during PTH1R–PTH complex internalization (in endosomes); (**b**) action of negative allosteric modulator Pitt12 on PTH1R inhibition of catabolic actions of PTH without deactivating anabolic ones [50]. Created in BioRender. BioRender.com/l16l797.

Allosteric modulators of PTH1R, such as Pitt12, may potentially modulate receptor activity in a way that achieves both antiresorptive and hypocalcemic effects while still preserving certain stimulatory effects of the receptor. In a study by Ieva Sutkeviciute et al. [50], it was demonstrated that the negative effect of Pitt12 on PTH1R stimulation by PTH primarily occurs through the inhibition of cAMP production from sources generated during PTH1R–PTH complex internalization (in endosomes), which is associated with cPTH stimulation and largely responsible for its catabolic effect. In contrast, cAMP production occurring at the cell membrane is moderately affected. This pathway is believed to be responsible for the transient stimulation of bone cells by PTH, which induces its anabolic effect, and is used in therapy. Targeting different molecular pathways induced by PTH–PTH1R binding can be responsible for the net effect on bone structure obtained during therapy (Figure 1). Consequently, allosteric modulators of PTH1R seem to be a potential drug in the treatment of PHPT-induced hypercalcemia and bone disease secondary to it, but it needs further research.

#### 3.1.2. RANKL/RANK/OPG

The primary molecular pathway responsible for the differentiation and activation of osteoclasts involves the production of RANKL by osteoblasts, which then binds to RANK on osteoclast precursors [24,25,29]. Additionally, osteocytes up-regulate RANKL expression in response to PTH stimulation, and this process is necessary in adult bone resorption [42,55].

**Figure 2:**
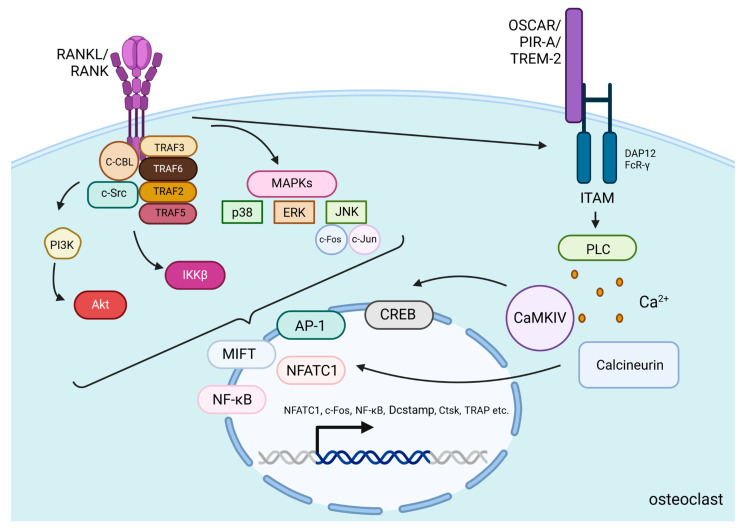
RANKL/RANK signaling pathways. RANKL/RANK binding induces the activation of TRAF proteins and the phosphorylation of c-Src by c-Cbl to stimulate PI3K/Akt, IKKβ, and MAPKs pathways [57,58,59]. These pathways alongside RANK-induced co-stimulatory calcium signaling from immunoglobulin-like receptors (OSCAR, PIR-A, TREM-2) associated with ITAM-containing adaptor proteins (DAP12 and FcR-γ) induce the translocation and activation of transcription factors including NFATc1, MITF, NFκB, AP-1, and CREB. High expression of these factors (especially NFATc1) in the nucleus is responsible for osteoclast-specific gene transcription [60,61,62]. Additionally, they increase their own production to create a stronger and more continuous signal in the cell. All of this accounts for osteoclast differentiation, activation and promotion of survival. Created in BioRender. BioRender.com/e03x846.

After PTH binds to PTH1R on the surface of osteoblasts and osteocytes, the Gαs/cAMP/PKA pathway is activated, leading to the phosphorylation of CREB, which then binds to specific DNA sequences to promote the transcription of RANKL. Then, the protein is presented on the cell surface or released as a soluble form and binds to RANK, which is located on the surface of osteoclast precursors and mature osteoclasts [56]. Cellular signals transducing after RANKL/RANK binding in osteoclasts involve the recruitment of many proteins called tumor necrosis factor receptor-associated factors (TRAF 2, TRAF3, TRAF5, and TRAF6), which are involved in osteoclast differentiation [57]. Especially activation of one of them—TRAF6—induces the c-Cbl phosphorylation by c-Src, which is essential in obtaining cytoskeletal organization, formation of the F-actin ring, and the resorptive function of osteoclasts [57]. Activation of those proteins induces stimulation of PI3K/Akt, IκB kinase β (IKKβ), and mitogen-activated protein kinases (MAPKs) pathways [57,58]. MAPKs include c-Jun N-terminal kinase (JNK), extracellular signal-regulated kinase (ERK), and p38 [59]. Those kinases phosphorylate several transcription factors that control osteoclastogenesis, including nuclear factor of activated T-cells cytoplasmic 1 (NFATc1), nuclear factor kappa-light-chain-enhancer of activated B cells (NF-κB), c-Jun, c-Fos, and microphthalmia-associated transcription factor (MITF) [60]. c-Fos and c-Jun dimerize to form the activator protein-1 (AP-1) complex, which, together with NF-κB, is necessary to induce the transcription of NFATc1—a protein that is a key regulator of osteoclastogenesis [61] (Figure 2).

The strong stimulation of NFATc1 protein production relies on RANKL-induced co-stimulatory calcium signaling. For efficient activation of Ca^2+^ signaling, immunoglobulin-like receptors (OSCAR, PIR-A, TREM-2) associated with ITAM-containing adaptor proteins such as DAP12 and FcR-γ are needed. Through the activation of Phospholipase C (PLC), Ca^2+^ is released into the cytoplasm and activates calcium/calmodulin-dependent protein kinase IV (CaMKIV). Then CaMKIV stimulates the transcription of CREB and enhances NFATc1 expression. This process is also related to increased c-Fos expression and higher AP-1 complex production. Both of the mentioned processes co-operate to increase NFATc1 levels [62]. Additionally, calcium signaling activates the phosphatase calcineurin, which facilitates NFATc1’s action in the nucleus [60]. In the nucleus, NFATc1, alongside AP-1, NF-κB, and other transcription factors, induce osteoclast-specific target genes responsible for the differentiation of osteoclast precursors into mature osteoclasts, activation of these osteoclasts, and promotion of their survival [60] (Figure 2).

RANKL expression can be influenced by other transcription factors, such as myocyte enhancer factor 2C (MEF2C). The promoter region of the RANKL gene contains three specific sites where the transcription factor MEF2C can bind. Before PTH stimulation, MEF2C is linked to histone deacetylase 4 (HDAC4), and this complex represses RANKL promoter activity. After PTH–PTH1R binding, the degradation of HDAC4 via the PKA-SMAD ubiquitination regulatory factor 2 (SMURF2) pathway is promoted, leading to the MEF2C release and increased RANKL production [63]. Additionally, MEF2C has been shown to promote RANKL-mediated induction of transcription factors c-Fos and NFATc1, leading to increased osteoclastogenesis [61]. In another study, MEF2C was demonstrated to play an important role in osteoclast differentiation into large multinuclear cells essential for effective bone resorption [64].

OPG is a glycoprotein and acts as a decoy receptor for RANKL, preventing RANKL from binding to its receptor, RANK, on the surface of osteoclast precursors. By inhibiting this interaction, OPG reduces the formation, function, and survival of osteoclasts. In PHPT, the level of OPG decreases [24,29]. PTH reduction in OPG gene transcription requires the involvement of CREB and AP-1 transcription factors [29]. In the study of bone biopsies in patients with PHPT and after PTX [65], it was shown that PTH has an important impact on the gene expression of RANKL and OPG, as evidenced by the decreased RANKL/OPG ratio after PTX. The balance between RANKL and OPG is crucial for maintaining bone density because a higher RANKL/OPG ratio (like in PHPT) promotes bone resorption, and on the contrary, a lower ratio favors bone formation [25,66].

As mentioned before, the molecule targeting the RANKL/RANK/OPG pathway is denosumab. This antiresorptive agent is recommended for patients with low BMD who are not candidates for PTX [17]. It was shown to reduce the intensity of bone remodeling in PHPT [36]. Studies involving denosumab treatment in PHPT patients have shown an increase in BMD at the hip region, lumbar spine, and distal radius [36,67]. Moreover, in the study by Daichi Miyaoka et al. [68], it was demonstrated that treatment with this monoclonal antibody was as effective as PTX in improving lumbar BMD and additionally enhanced lumbar spine TBS. However, the increase in total hip BMD was not as significant as in the PTX group. In a study by Cristina Eller-Vainicher et al. [69], 2 years of denosumab treatment demonstrated greater potency in increasing bone mass at the femoral and lumbar sites in older women with PHPT compared to those with postmenopausal osteoporosis. This may reflect the heightened activation of the RANKL/RANK pathway in PHPT, which could explain the superior therapeutic effects achieved by its inhibition in this patient group. However, as noted earlier, denosumab lacks significant bone-building abilities, and treatment with this agent cannot fully restore already lost bone mass.

#### 3.1.3. Interleukin-17A (IL-17A)

There is growing evidence that elevated levels of PTH lead to low-grade inflammation, as it was evaluated by measurement of pro-inflammatory cytokines like interleukin-6 (IL-6), interleukin-1 (IL-1), tumor necrosis factor alpha (TNF-α), and interleukin-17A (IL-17A) [55,57,70,71,72]. These cytokines were demonstrated to play a significant role in enhancing the expression of RANKL, positively correlate with BTM levels and the intensity of bone resorption [55,73], and may be involved in bone loss in patients with PHPT. T lymphocytes express PTH1R on their surface, and cPTH stimulates them to TNF-α production [74]. TNF-α promotes the CD4+ cell differentiation into Th17 cells, which synthesize IL-17A. This process is also stimulated by IL-6 and transforming growth factor beta (TGF-β) [75], which are produced by osteoblasts and osteocytes during cPTH exposure [76]. IL-17A intensifies the response of osteoblasts and osteocytes to PTH, thereby increasing the production of RANKL [55,73] and enhancing osteoclastogenesis. IL-17A was also demonstrated to potentiate the osteoclastogenic activity of RANKL by up-regulating RANK on osteoclast precursors [77].

Jau-Yi Li et al. showed that IL-17A levels are elevated in PHPT, but they decrease following PTX [78]. Additionally, they showed that in wild-type mice treated with cPTH, the addition of neutralizing antibody directed against murine IL-17A (IL-17A Ab) caused a reduction in bone resorption, while bone formation was not affected. MicroCT analysis showed that these mice did not experience a reduction in cortical bone thickness (Ct.Th) and volume (Ct.Vo) nor in trabecular bone volume (BV/TV), while in mice treated with cPTH and an irrelevant antibody, bone loss was demonstrated. The preventive role of IL-17A inhibition was confirmed in more detailed trabecular bone structure analysis, which showed trabecular number (Tb.N) and trabecular space (Tb.Sp) preservation in mice treated with IL-17A Ab. However, regardless of the antibody used, cPTH reduced trabecular thickness (Tb.Th) in all mice. Histomorphometric analysis of the femur’s cancellous bone showed that mice treated with the IL-17A Ab had a lower number of osteoclasts per bone surface (N.Oc/BS) and a smaller percentage of surfaces covered by osteoclasts (Oc.S/BS). Additionally, IL-17A Ab treatment did not affect bone formation increased during cPTH stimulation assessed by mineral apposition rate (MAR) and bone formation rate (BFR). BTMs measurements also confirmed decreased bone resorption and preserved bone formation during the treatment with IL-17A Ab, as the increase in C-terminal telopeptide of type I collagen (CTX) level caused by cPTH was significantly reduced while the increase in Procollagen Type 1 N-Terminal Propeptide (P1NP) level was not affected.

Taking into consideration that PHPT can be perceived as a low-inflammatory disease, the introduction of anti-inflammatory treatment could bring many beneficial effects in PHPT-related bone disease. Particular attention is paid to IL-17A inhibitors, which could be effective in decreasing RANKL-mediated osteoclastogenesis and bone resorption. Nowadays, IL-17A inhibitors like secukinumab, ixekizumab, and brodalumab are used for psoriasis, psoriatic arthritis, and ankylosing spondylitis [79]. In the above-mentioned study by Jau-Yi Li et al. [78], it was shown that IL-17A inhibitors are effective in preventing cortical bone loss typical in PHPT and preserving proper bone architecture. Additionally, they were shown not to affect PTH-mediated increases in bone formation. These effects make them a very interesting potential method of treatment in this indication, as they could potentially improve bone structure, which is important in maintaining bone strength. The application of them in bone disease secondary to PHPT needs further research as their usage is not yet established in clinical practice.

#### 3.1.4. Macrophage Colony-Stimulating Factor (M-CSF)

M-CSF, also known as CSF-1, is indispensable during the RANKL/RANK interaction [80]. PTH is one of the factors that induces the production of M-CSF by osteoblasts/bone marrow stromal cells and cytokines such as TNF-α and IL-1 can enhance it [80]. In PHPT patients, M-CSF is significantly up-regulated. M-CSF binds to colony-stimulating factor receptor 1 (CSF1R) on macrophage/monocyte precursors and activates c-src/PI3K/Akt and ERK/MAPK signaling pathways [81], which initiates the proliferation, differentiation, and survival of the osteoclast precursors and increases the expression of RANK on their surface [80]. Preosteoclasts form after RANKL/RANK binding, and then they differentiate and fuse into mature, multinucleated bone-resorbing osteoclasts. Stimulation of CSF1R by M-CSF on mature osteoclasts enhances their size and resorption activity [28] and plays an important role in cytoskeletal reorganization and cell migration [82]. To sum up, M-CSF signaling is essential for osteoclast survival, function, proliferation, and differentiation.

Blockade of M-CSF or CSF1R could be a potential target for new drugs in bone loss. In ovariectomized mice, bone loss, increased osteoclast number due to estrogen deficiency, and up-regulated M-CSF production were terminated after treatment with anti-M-CSF antibody [83]. Reduction in the osteoclasts number and increased bone volume in microCT scans were observed, especially in osteoporotic female mice treated with anti-CSF1R antibody, which suggests this antibody could protect from age-related bone loss [84]. Anti-CSF1R antibodies also decreased bone resorption and osteoclast formation in ovariectomized mice [85]. Treatment with another anti-CSF1R antibody reversed trabecular and cortical bone degradation in mice with secondary osteoporosis due to type I diabetes mellitus [86]. To date, there is no data regarding the use of M-CSF, CSF1R antibodies, or inhibitors in PHPT-related bone disease, although the effects observed in the above-mentioned studies indicate that there is a high chance of a positive outcome of such therapy.

#### 3.1.5. Monocyte Chemoattractant Protein-1 (MCP-1/CCL-2)

Besides RANKL/RANK and M-CSF, PTH induces, and cPTH up-regulates, the production of a chemokine called monocyte chemoattractant protein-1 (MCP-1/CCL-2) which is crucial for PTH-induced bone loss [83] and is up-regulated in cases of cPTH stimulation [44,87,88,89]. Enhanced synthesis of this chemokine in PHPT is associated with induced osteoblastic secretion after PTH-PTH1R binding [83,89]. Additionally, osteoclasts themselves can further secrete MCP-1, contributing to the process of RANKL-induced osteoclastogenesis through autocrine/paracrine signaling [90]. To obtain the ability to resorb, osteoclasts require the presence of RANKL [91]. RANKL induces production of MCP-1 and its receptors (chemokine receptor 2 and chemokine receptor 4) during osteoclast differentiation [91]. MCP-1 binds to its primary receptor, chemokine receptor 2 (CCR-2), present on osteoclast precursors and mature osteoclasts and participates in monocyte recruitment, chemotaxis, and osteoclast differentiation [28,91]. MCP-1 increases the number of RANK presented on osteoclasts [92] and enhances the RANKL-mediated osteoclastic bone resorption [89]. Furthermore, MCP-1 mediates the inhibition of granulocyte-macrophage colony-stimulating factor (GM-CSF) secretion, which is known to reduce osteoclastogenesis [91]. MCP-1 is up-regulated by M-CSF. On the other hand, MCP-1 is a critical regulator of M-CSF actions in bone remodeling: it enhances the expression of the CSF1R gene and activates key signaling pathways involved in the formation and function of osteoclasts and in cytoskeletal organization and actin ring formation by affecting c-src/PI3K/Akt and ERK/MAPK signaling pathways and small GTPase activation (Rho and Rac1) [92].

In a study by Jawed A. Siddiqui et al. [83], it was demonstrated that in MCP-1 knockout mice with a murine model of PHPT, PTH-induced characteristic cortical and trabecular bone loss was prevented. This finding provides evidence for the essential role of MCP-1 in cPTH catabolic effects and bone resorption observed in this condition. MCP-1 was found to be essential for maintaining the PTH-induced recruitment of monocytes, macrophages, and osteoclasts in bone and the formation and function of the latter. Additionally, in the OK-JOO SUL et al. study [92], it was observed that in mice lacking MCP-1, the femur BMD was increased, and greater trabecular bone volume (BV/TV), more trabeculae (Tb.N.), and less separation between trabeculae (Tb.Sp.) in microCT were noted. However, this positive effect on bone formation was not present in the previously mentioned study. In the study by Morrison et al. [93], an MCP-1 inhibitor (7ND) added to osteoclast cultures containing RANKL and M-CSF blocked the osteoclast differentiation, highlighting the important role of MCP-1 in osteoclastogenesis. All of these findings together underline the critical role of MCP-1 in osteoclast differentiation and bone resorption.

Both inhibiting the M-CSF/CSF1R pathway and MCP-1 activity can result in decreased osteoclastogenesis and, in turn, reduced bone turnover. Therefore, the antiresorptive effect thus obtained is comparable to that caused by bisphosphonates or denosumab, which are approved drugs in treatment of bone loss secondary to PHPT [17]. Those agents were shown to enhance bone mineralization and prevent further bone microarchitecture deterioration in the trabecular site (by preventing the loss of trabecular number and thickness) and cortical site (by reducing cortical porosity), although their abilities to restore the already lost bone microarchitecture are limited [32]. Inhibition of both molecular pathways mentioned above seems to improve cortical and trabecular bone generally without impact on bone formation and can act as an alternative to currently used drugs. To evaluate if they have any advantages over the main presently used antiresorptives, especially in PHPT, needs further research.

### 3.2. Molecular Mechanism Involved in Increased Osteoclast Resorptive Functions in PHPT

Osteoclasts are formed from hematopoietic stem cells (HSCs), which are present in bone marrow (BM). Osteoclasts are derived from the monocyte–macrophage lineage. Bone marrow cavities create a unique microenvironment, known as a “niche”, which supports HSCs. These niches are made up of different types of cells, including osteoblasts, endothelial cells, and reticular cells [94]. Mature osteoclasts migrate to resorption sites. This results in conformational changes in these cells and the formation of specialized structures such as the ruffled border and actin ring (to create a sealing zone), which are essential for osteoclastic resorptive activity. Moreover, activated osteoclasts induce the transcription of proteases like cathepsin K (CTSK) and metalloproteinases, which break down the bone matrix. These enzymes require an acidic environment to function properly. This is facilitated by increased production of V-ATPases (the proton pumps), which appear on the ruffled border where they release H+ ions into the resorptive lacuna. In this separated and acidic environment, crystalline hydroxyapatite dissolves, allowing proteases to reach and degrade the bone matrix [95].

Inhibiting osteoclast resorptive activity in patients with PHPT seems to have the potential to improve bone mineral density and microstructure, despite high PTH levels. Disruption of primarily osteoclast functions without affecting their differentiation and quantity might preserve interactions between osteoclasts and osteoblasts; thus, substances produced by osteoclasts that stimulate osteoblasts are able to maintain their role [96]. As a result, bone remodeling and bone formation processes are not fully inhibited. This potentially generates the possibility of creating a therapy that will combine several desired therapeutic effects in the treatment of patients with bone loss: reduction in resorption and concomitant restoration of bone mass through maintained osteoblastic activity.

**Figure 3:**
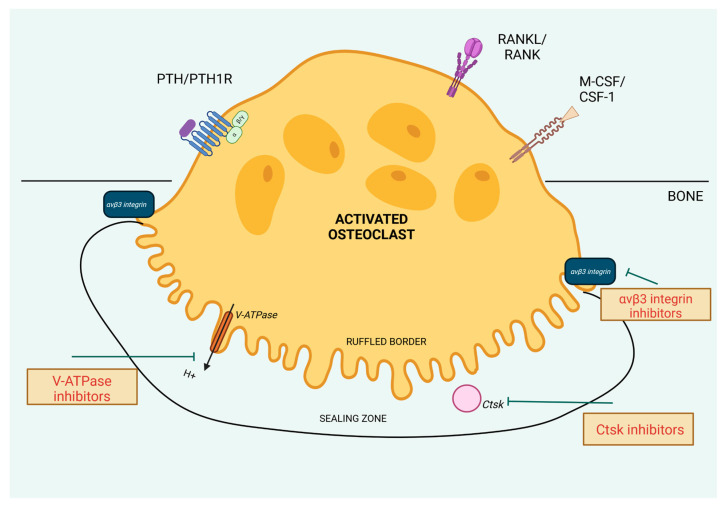
Molecular targets aimed to decrease osteoclast activity. Created in BioRender. BioRender.com/o84i540.

In this section, we will describe the molecular mechanisms that could be the targets to decrease the osteoclast activity (Figure 3). Many of them are up-regulated by cPTH stimulation, so halting them by specified molecules could provide precise treatment of bone loss in PHPT.

#### 3.2.1. αvβ3 Integrin

The integrins are specified transmembrane receptors, composed of alpha and beta subunits, which mediate cell–cell and cell–matrix interactions. These integrin-mediated adhesive interactions play an important role in bone resorption [97]. The αvβ3 integrins are abundantly present in osteoclasts, and they recognize and bind to the RGD (Arg-Gly-Asp) motif found in bone-specific proteins such as osteopontin and bone sialoprotein and also vitronectin, all of them found in bone tissue [97]. These integrins, together with M-CSF/CSF1R activation, are involved in osteoclast adhesion and cytoskeletal reorganization required for cell migration, polarization, and formation of the sealing zone, actin ring, and ruffled border, which are necessary for the resorption process [98,99,100]. The molecular pathway to achieve these osteoclast functions includes the actions of tyrosine kinase c-Src. It is a critical player in osteoclasts, contributing to the dynamic assembly and disassembly of podosomes, which are integrin-based structures that enable cell adhesion and movement. After αvβ3 integrin binding to extracellular matrix proteins, c-Src can interact with kinase Pyk2 and Cbl proteins to recruit other signaling molecules such as phosphatidylinositol 3-kinase (PI3K) and dynamin [98]. Additionally, c-Src can collaborate with kinase Syk, which is activated by ITAM-containing adapters like Dap12 and FcRγ. The c-Src–Syk complex activates downstream effectors, including Vav3, which induces the activity of the small GTPase Rac1, an important enzyme involved in cytoskeletal reorganization [99,100]. This reorganization also involves Rho, although Rho’s activation appears to be independent of Vav3 stimulation [100].

Antibodies targeting αvβ3 integrin, disintegrins, and molecules containing RGD motifs have been used as therapeutic targets as they were shown to impact the resorptive abilities of osteoclasts. However, integrins are involved in a wide range of physiological processes beside bone health. They also play key roles in cell adhesion, migration, immune responses, and regulation of hemostasis. For example, RGD motifs are present on fibrinogen and fibronectin and interact with αIIbβ3 integrin, the most important integrin for platelet aggregation and blood clot formation, which is structurally similar to αvβ3. Additionally, αvβ3 integrin is also found on platelets, although their role in clot formation is minimal [101]. To avoid unintended effects on hemostasis and achieve antiresorptive effects on bone, while developing therapeutic agents targeting RGD-binding integrins, the molecules should be highly specific and inhibit only the action of αvβ3 integrin.

The integrin αvβ3 is needed for PTH-induced osteoclastic activity to increase serum calcium, largely derived from bone resorption. McHugh et al. demonstrated that in β3 knockout mice osteopetrosis developed over time due to alteration in osteoclast resorptive function even though there was an increased number of osteoclasts in these mice [102]. Tzu-Hung Lin et al. [103] invented a protein derived from a modified version of the rhodostomin protein, combined it with human serum albumin to extend its duration of action and minimize immune reactions, and called it HSA-ARLDDL. This protein proved to be a potent αvβ3 antagonist, effectively inhibiting trabecular bone loss in ovariectomized mice and selectively blocking the fusion of osteoclast precursors into mature osteoclasts. Moreover, HSA-ARLDDL specifically inhibited αvβ3 integrin without affecting other integrins involved in platelet aggregation. Yuval Zur et al. [104] engineered bispecific M-CSF mutants (M-CSFRGD) that can bind both CSF1R and αvβ3 integrin. M-CSFRGD effectively inhibited osteoclast differentiation and activity and actin ring formation.

These substances require further research, both in terms of safety and specific effects on bones, as well as in the context of their use in PHPT.

#### 3.2.2. V-ATPase

When osteoclasts are activated, they adhere to the bone surface and become polarized to reorganize the cytoskeleton to form a sealing zone and ruffled border [95]. Cytoskeletal rearrangements are crucial in the trafficking and assembly of enzymes involved in bone resorption to the ruffled border, which is a specialized membrane domain. It faces the bone surface and is responsible for the acidification of extracellular space formed by sealing zones and the release of enzymes involved in bone resorption. The acidification process is maintained by specialized structures called V-ATPases. Once activated, V-ATPase pumps protons (H+) into the resorption lacuna, creating an acidic environment that is necessary for dissolving the bone mineral—hydroxyapatite—and for activating enzymes involved in degradation of the organic matrix of bone [95].

The V-ATPase is composed of two main domains: the peripheral V1 domain and the integral V0 domain. The V1 domain is responsible for ATP hydrolysis, which provides the energy required for proton pumping, and the V0 domain is responsible for proton translocation across the membrane. The V1 components include A-H subunits, whereas V0 consists of a, c, c′′, d, and e subunits [105]. Each subunit plays a distinct and essential role within its domain. Specified interactions between subunits are critical to ensuring the overall activity of the enzyme complex [106,107]. Three specific parts of V-ATPases involved in bone resorption—B2 isoform of the B-subunit, the a3-isoform of the a-subunit, and the d2-isoform of the d-subunit—were demonstrated to be crucial in the catabolic activity of osteoclasts [106,108].

Due to its critical role in the resorption process, V-ATPase inhibition could serve as a therapeutic target for treating bone loss.

Bafilomycins, the macrolide antibiotics, inhibit V-ATPase by binding to its V0 domain and reducing proton translocation. They have been widely used in experimental conditions to study V-ATPase function but have limited clinical use due to toxicity.

Osteoclast-specific inhibitors of V-ATPase were created, such as SB 242784 or FR167356, which reduce bone resorption and prevent bone loss in rat models [109,110]. FR167356 was shown to selectively reduce the activity of V-ATPase in osteoclasts without significant effects on murine lysosomal V-ATPase. This molecule also inhibited PTH-induced calcium release associated with bone resorption.

Luteolin, which is a flavonoid, specifically inhibits the binding between a3 and d2 V-ATPase subunits characteristic for osteoclast. Consequently, it interrupts the acidification process in osteoclasts and reduces their bone-resorbing activity without affecting osteoclast proliferation, viability, or morphology [106].

Enoxacin, a fluoroquinolone antibiotic, inhibits the formation and function of osteoclasts by interrupting the interaction between the V-ATPase B2 subunit and cell microfilaments. However, large doses of enoxacin are needed to observe the therapeutic effect, which is associated with several side effects. To improve the safety of therapy and achieve high accumulation of enoxacin in bone, it was conjugated with bisphosphonate to form bis-enoxacin (BE). Quiank Xu et al. demonstrated that BE effectively inhibited osteoclast proliferation, differentiation, and function. It disturbed osteoclast actin ring formation and bone resorption [111]. In the rat model of postmenopausal osteoporosis, BE has shown promising results in improving trabecular bone microarchitecture [111], cortical bone thickness and strength, and reducing cortical porosity [112]. It was also demonstrated that rats treated with bis-enoxacin exhibited better biomechanical features and higher bone strength than the controls treated only with bisphosphonate (zoledronate) [112]. Moreover, bis-enoxacin treatment appears to be a safe treatment option [111,112].

Osteoclast V-ATPase expression and bone resorption activity are enhanced by the PTH-PTH1R binding [46]. In PHPT, this process probably intensifies, leading to increased bone resorption. Bis-enoxacin and other V-ATPase inhibitors have not been specifically studied in PHPT. Luteolin, SB 242784, or FR167356 decreased bone resorption without affecting the number or morphology of osteoclasts and, in turn, did not disturb osteoclast–osteoblast interactions during bone remodeling. Bis-enoxacin, the potent molecule inhibiting both osteoclast proliferation and activity and bone resorption, was shown to improve the cortical bone, which is highly affected in patients with PHPT. Although, by binding with bisphosphonate, it obtained the property to increase osteoclast apoptosis, consequently osteoclast–osteoblast interactions could be affected during its usage.

#### 3.2.3. Cathepsin K (CTSK)

The main resorptive enzymes produced by osteoclasts after the formation of the ruffled border and actin ring are CTSK and matrix metalloproteinase-9 (MMP-9). Their role is to degrade the extracellular bone matrix, especially collagen fibers. They are activated in the acidic environment created by the V-ATPases. Expression of both enzymes is enhanced in PHPT [113]. CTSK is a major collagen-degrading protease in osteoclasts, crucial for bone resorption. Its expression is selectively high in osteoclasts, where it is controlled by RANKL, which activates NFATc [114]. CTSK is also responsible for decreased bone formation of cortical bone in response to mechanical loading because of its ability to degrade the protein called periostin (POSTN).

POSTN is a matricellular protein highly secreted by osteocytes and periosteal osteoblasts [115,116]. Its primary roles are the creation and organization of fibrils in the extracellular matrix, which provides structural support and strength to tissues. Additionally, as a ligand for integrins αvβ3 and αvβ5, POSTN regulates cell adhesion and motility [117]. POSTN expression is up-regulated by mechanical loading and exercise, playing a crucial role in determining bone mass and microstructure in response to such stimuli. This effect is particularly significant in the periosteal compartment, where it enhances cortical bone mass, thickness, and strength [117], thereby protecting against fractures and supporting fracture healing [118].

The molecular mechanism responsible for the anabolic effects of POSTN involves the decrease in sclerostin levels, which in turn activates the WNT/β-catenin signaling pathway to induce osteoblast actions on bone [118]. That pathway is also responsible for the anabolic effects of PTH stimulation on cortical bone [119], as PTH increases POSTN expression [29,113]. In the absence of POSTN, bone formation is impaired, and PTH cannot effectively improve cortical structure and strength [119].

The increased expression of CTSK observed in PHPT patients leads to enhanced POSTN degradation and high levels of its product, K-perostin, which can be measured in blood. In the study by J. Pepe et al. [120], K-periostin was found to be an independent marker of incident fractures in postmenopausal women with PHPT, in the absence of significant differences in BMD and BTMs values between the fracture and non-fracture groups. Inhibition of CTSK was shown to increase the level of periostin and preserve or even increase bone formation [121].

Targeting CTSK is emerging as a highly effective treatment approach for managing bone loss in PHPT. CTSK inhibitors specifically block its collagenase activity and reduce bone resorption without impairing osteoclast differentiation, migration, or survival. As a result, osteoclasts still express molecules that promote osteoblast differentiation at remodeling sites [96] and preserve their essential functions to maintain bone health and enable bone regeneration [122].

Unfortunately, CTSK active site inhibitors such as relacatib, balicatib, and odanacatib have been found to cause serious side effects and have not been approved for treatment. There is ongoing research exploring a method called ectosteric protease inhibition to selectively block CTSK’s collagenase activity by targeting specific external sites, leaving its active site and other functions intact [123]. This approach has shown potential with fewer side effects compared to traditional inhibitors. The use of CTSK inhibitors in bone loss secondary to PHPT seems to be a promising therapy, especially due to the maintenance of bone formation and significant impact on the reconstruction of the cortical bone and its strength.

### 3.3. Changes in Levels of Wnt/β-Catenin Pathway Inhibitors in PHPT and Its Effect on Bone Formation

The Wnt/β-catenin pathway, also known as the canonical Wnt signaling pathway, is an important regulator of various cellular processes, including cell proliferation, differentiation, migration, and survival. It plays a particularly significant role in bone metabolism. Wnt proteins are a family of secreted glycoproteins that initiate the Wnt/β-catenin signaling pathway. Wnt ligands bind to cell surface receptors of the frizzled family (FZD) and co-receptors called low-density lipoprotein receptor-related proteins 5 and 6 (LRP5/6). In the absence of Wnt ligands, β-catenin is phosphorylated by a destruction complex (consisting of Axin, adenomatous polyposis coli, casein kinase 1, and glycogen synthase kinase 3β) and undergoes ubiquitination and proteasomal degradation, which prevents it from entering the nucleus. When Wnt ligands bind to FZD and LRP5/6 receptors, the destruction complex is inhibited. This stops β-catenin phosphorylation, leading to its stabilization and accumulation in the cytoplasm. Stabilized β-catenin translocates to the nucleus, where it interacts with T-cell factor/lymphoid enhancer factor (TCF/LEF) transcription factors. This interaction activates TCF/LEF and triggers the transcription of Wnt target genes involved in osteoblast proliferation, differentiation, and survival [124]. Activation of the Wnt/β-catenin pathway also impacts osteoclast production because β-catenin, in conjunction with TCF proteins, induces osteoblast expression of OPG [125]. PTH binding to PTH1R can initiate this pathway without Wnt ligands by forming a complex with LRP5/6, which prevents β-catenin phosphorylation and leads to its action [126].

#### 3.3.1. Sclerostin (SOST)

**Figure 4:**
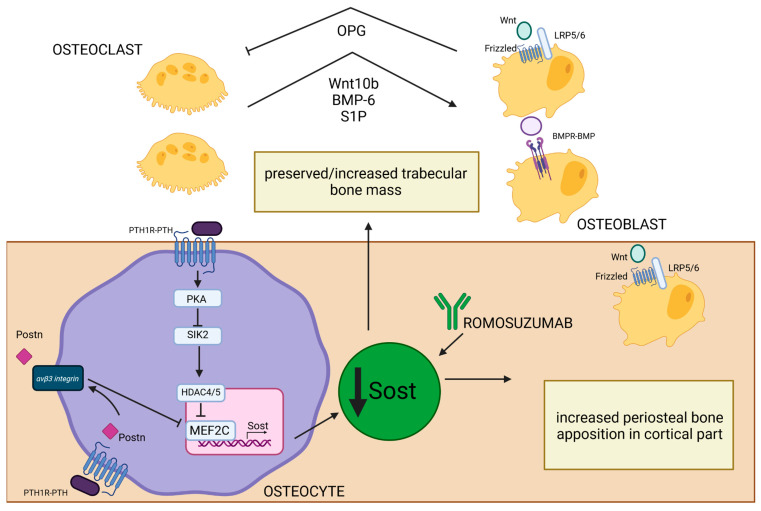
Effect of PTH-induced SOST reduction on bone structure in PHPT. PTH1R–PTH binding on the surface of osteocytes leads to PKA activation and SIK2 inhibition. Inactivation of SIK2 permits the HDAC4/5 migration into the nucleus, where it creates a complex with MEF2C—a crucial transcription factor in regulating the expression of gene-encoding SOST [128]. Low SOST concentrations lead to increased osteoblastogenesis through the activation of Wnt/B-catenin and BMPR-BMP pathways [127,129,130]. In trabecular bone, this process is enhanced by increased synthesis of Wnt10b, BMP-6, and S1P by osteoclasts in remodeling sites [132]. Activated osteoblasts, through the Wnt/B-catenin pathway, can increase the OPG level and influence osteoclast resorptive activity [125]. All of those processes may be responsible for preserved/increased trabecular bone mass observed in PHPT. MEF2C and SOST transcription could also be inhibited by PTH-induced POSTN synthesis, and this process occurs through the integrin αVβ3 receptor [119]. This is especially important in the periosteal part of cortical bone, where low levels of sclerostin permit increased periosteal osteoblast activation through Wnt/β-catenin pathway and can explain the enhanced periosteal apposition in cortical bone. The similar effect of SOST inhibition could be achieved by romosozumab—a monoclonal antibody that selectively blocks SOST [135,136]. Created in BioRender. BioRender.com/u55g815.

Sclerostin is a glycoprotein primarily produced by osteocytes [127] whose expression is controlled by PTH [27]. After PTH-PTH1R binding on the surface of osteocytes, activated PKA inhibits salt-inducible kinase 2 (SIK2), whose role is to phosphorylate HDAC4/5. Inactivation of SIK2 permits the HDAC4/5 migration to the nucleus, where it creates a complex with MEF2C, a transcription factor crucial in regulating the expression of gene-encoding sclerostin (SOST). HDAC4/5–MEF2C binding represses the transcription of SOST [128]. SOST is an inhibitor of the Wnt signaling pathway and negatively regulates bone formation. It binds to the LRP5/6 co-receptors, preventing the activation of Wnt target genes, which are essential for osteoblast differentiation and activity [129]. SOST is also the potent antagonist of bone morphogenetic proteins (BMP), which are a group of growth factors engaged in the process of differentiation of mesenchymal stem cells into osteoblasts [127,130]. Studies have shown that PTH suppresses SOST expression [27,128,131] and that sclerostin level negatively correlates with PTH and calcium concentration in PHPT [131]. Reducing the inhibitory effects of sclerostin on BMP and Wnt by PTH [27] leads to enhancement of bone formation. It is thought to be a mechanism to compensate for increased bone resorption as an attempt to maintain bone remodeling balance in PHPT patients. Mature osteoclasts were shown to have a crucial role in this process because they can induce bone formation by enhanced synthesis of BMP-6 and Wnt10b in remodeling sites and activate Wnt and BMP signaling pathways in mesenchymal stem cells. Additionally, they can recruit osteoprogenitors to remodeling sites by BMP-6 and increased production of sphingosine kinase 1 (SPHK1), which increase the level of a signaling molecule called sphingosine 1-phosphate (S1P) and promote chemokinesis [132]. Those osteoclast–osteoblast interactions are present mainly in parts of bone with increased remodeling processes, such as the trabecular compartment, and those interactions alongside the reduction in sclerostin expression by trabecular osteocytes may explain preserved or sometimes even increased bone mass in calcaneus bone observed in PHPT [18]. Conversely, in the endosteal part of cortical bone, which is characterized by a high remodeling rate, the mentioned actions do not prevent increased cortical porosity typical for cPTH stimulation [18]. Therefore, even increased Wnt signaling is not sufficient to suppress destruction of the endosteal surface associated with increased osteoclast-induced enhanced resorption [133]. In the periosteal part of cortical bone, where modeling-based formation is mainly present, the cPTH stimulation also contributes to decreased sclerostin expression by periosteal osteocytes and, in turn, enhanced activation of Wnt/β-catenin signaling [134]. Furthermore, POSTN was shown to be involved in PTH-induced SOST inhibition [119]. Activation of the Wnt/β-catenin pathway may be responsible for compensatory periosteal bone apposition observed in PHPT [58]. Unfortunately, this process cannot be fully efficient due to decreased proliferation and differentiation of osteoblasts in the periosteum caused by cPTH stimulation. Additionally, increased osteoclast level in the endosteal part of cortical bone makes it even more impossible to fully counterbalance the loss of cortical bone mass and structural integrity. The net effect of cPTH stimulation is decreased cortical thickness observed in PHPT. This imbalance contributes to overall bone weakening and an increased risk of fractures (Figure 4).

Romosozumab is a monoclonal antibody that targets and inhibits SOST (also known as Scl-Ab) (Figure 4), so it increases the activity and number of osteoblasts, leading to increased bone formation [135,136]. Additionally, due to increased Wnt signaling, it also prevents bone resorption (likely by promoting OPG production) [126,135,136]. This results in decreased bone turnover, increased BMD, and reduction in fractures in osteoporotic postmenopausal women [136]. In the Pascale Chavassieux et al. study [137], treatment of postmenopausal osteoporotic women with romosozumab caused increased bone formation and decreased bone resorption, especially in the trabecular and endocortical compartments, and an overall increase in bone mass and thickness evaluated by histomorphometric studies of bone biopsies. This antibody was shown to increase bone formation in areas of high remodeling (especially trabecular and endocortical surfaces), but also on quiescent (inactive) bone surfaces undergoing modeling-based formation [136,138].

The results of these studies suggest that the increased remodeling with predominant resorption observed in PHPT could probably be effectively counteracted by romosozumab. Furthermore, Scl-Ab may amplify the PTH-mediated reduction in sclerostin levels, thereby enhancing the Wnt/β-catenin signaling pathway and promoting osteoblastogenesis. To date, there are no studies investigating the use of romosozumab in treating bone loss specifically due to PHPT. However, two case reports presented at the 26th European Congress of Endocrinology in Stockholm discussed the treatment of PHPT patients with secondary osteoporosis using romosozumab following PTX. In both cases, a significant increase in BMD, particularly in the lumbar spine, was observed after one year of treatment with romosozumab [139].

#### 3.3.2. Dickkopf-1 (DKK-1)

Dickkopf-1 (DKK-1) is another significant inhibitor of the Wnt signaling pathway. It binds to LRP5/6 co-receptors, preventing Wnt ligands from activating their signaling pathways [140,141]. In addition to LRP5/6, DKK-1 requires binding to Kremen1/2 (KRM-1 and KRM-2) to mediate the internalization and degradation of the entire complex and consequently to suppress further canonical Wnt pathway signaling [142]. DKK-1 inhibits the Wnt signaling pathway similarly to SOST, suppressing osteoblast activity and bone formation. Studies in mice demonstrated that a gradual decrease in DKK1 resulted in increased trabecular and cortical bone mass, while the increase in DKK1 led to bone loss [138].

The relationship between DKK-1 and PTH in PHPT is complex and less well understood compared to SOST. Kulkarni et al. [143] investigated the effects of cPTH exposure on the WNT/β-catenin pathway on rat distal metaphyseal bone in vivo and rat osteoblastic osteosarcoma cells (UMR 106) in culture. They demonstrated that PTH increases the expression of the FZD-1 receptor and decreases the expression of DKK-1, leading to enhanced Wnt signaling. Although PTH also increases the expression of KRM-1, in the absence of DKK-1, KRM-1 alone cannot effectively inhibit the pathway. Jun Guo et al. [144] confirmed decreased levels of DKK-1 in three distinct models of hyperparathyroidism. In genetically modified mice overexpressing DKK-1, compared to wild-type mice, the PTH-induced increase in the number of osteoblast precursors and bone formation were inhibited. Although, despite DKK-1 overexpression, PTH was shown to still increase Wnt/β-catenin signaling by cooperating with other Wnt ligands (such as Wnt3a), reducing the level of SOST, and promoting PKA-mediated phosphorylation of β-catenin. The study suggests that inhibiting DKK-1 alone may not sufficiently counteract the effects of chronic PTH stimulation. Conversely, studies on long-term treatment with teriparatide have shown that after more than 12 months of treatment, the levels of DKK-1 begin to increase. The rise in DKK-1 levels may explain the diminishing effects of this therapy in postmenopausal osteoporosis. It has been suggested that chronic PTH stimulation of the osteoblast lineage might trigger a homeostatic reaction, down-regulating its anabolic effects through an oversecretion of DKK-1 [145]. In a study of PHPT patients, DKK-1 levels were significantly higher than in the control group, with concomitant decreased SOST levels [146]. Considering the divergent effects of cPTH stimulation on DKK-1 expression, it is difficult to clearly determine the role of DKK-1 in the development of bone disease in PHPT. Reduced DKK-1 concentrations observed in some studies could explain the enhanced activity of osteoblasts in response to PTH, the increase in bone formation, and elevated concentrations of bone formation markers (e.g., P1NP). On the other hand, considering that long-term PTH stimulation can lead to a gradual increase in the amount of DKK-1, it cannot be ruled out that this is a factor that leads to the escalation of the predominance of resorption processes over bone formation, which will ultimately lead to the intensification of clinical manifestations of the disease. Considering the latter theory, treatment aimed at down-regulating the DKK-1 levels could enhance osteoanabolic properties of PTH by increased stimulation of the Wnt/β-catenin pathway.

Monoclonal antibodies against DKK-1 (DKK1-Ab) have been developed and evaluated in various animal models to assess their impact on bone mass [138]. DKK1-Ab was shown to increase bone formation and BMD in gonad-intact rodents, particularly in growing ones [147]. In ovariectomized mice with estrogen-deficiency-induced osteopenia, 8 weeks of DKK1-Ab treatment restored BMD in the femur and lumbar spine to levels observed in sham-operated controls. Furthermore, DKK1-Ab exhibited significant bone-anabolic activity in aged and ovariectomized nonhuman primates, increasing BMD in whole-body and spine evaluated by longitudinal DXA, and improving bone microarchitecture demonstrated by HR-pQCT analysis of the distal tibia and radius [148]. However, according to another study, DKK1 plays a limited role in adult rats with estrogen deficiency, as DKK1 inhibition did not significantly affect DXA BMD measurements at the lumbar spine (L1–L5) or femur-tibia [147]. DKK1-Ab was also found to block systemic inflammation-related bone erosion and promote bone formation in rheumatoid arthritis. Additionally, DKK-1 blockade reduced osteoclastogenesis by increasing OPG production. In this case, DKK-1 was up-regulated by high TNF levels, which supports the hypothesis that inflammation significantly induces DKK-1 expression, linking the immune system to bone formation [149,150]. This is particularly relevant since PHPT is also considered an inflammatory disease; although recent studies on the use of DKK1-Ab in this condition are lacking, further research is needed.

## 4. Materials and Methods

Firstly, databases such as PubMed, Science Direct, and Scopus were searched to identify studies that describe the impact of PHPT on bone metabolism and bone structure. Keywords used included “primary hyperparathyroidism”, “PHPT” in combination with “bone”, “bone metabolism”, “osteoporosis”, “bone loss”, “bone resorption”, as well as “RANKL/RANK/OPG” and “Wnt/β-catenin pathway”. The selected studies highlighted significant bone metabolic changes characteristic of PHPT, as well as those that explored potential treatments aimed at reversing the metabolic abnormalities associated with excessive PTH production, which lead to pathological bone remodeling. Studies conducted in animal models or in vitro, along with reviews and clinical trials, were included. Articles published since the year 2000 and written in English were considered.

## 5. Conclusions

The continuous stimulation of bone cells by PTH in PHPT leads to both anabolic and catabolic processes within the bone and to increased bone turnover, resulting in a predominance of resorptive processes, particularly causing bone loss in the cortical area and increasing the risk of fractures.

The primary treatment for PHPT-related bone disease is surgery, which is not always suitable for all patients, especially the elderly ones with other comorbidities. Moreover, PHPT is often mild and asymptomatic, with bone loss (sometimes of complex origin) being the only clinical manifestation that requires alternative treatment methods.

Currently available therapies, such as bisphosphonates and denosumab, effectively reduce osteoclast activity and preserve bone mass and structure. However, these treatments primarily target bone resorption and do not promote bone formation, potentially limiting their long-term efficacy due to the suppression of bone remodeling. Therefore, there is a need for therapies that limit resorptive processes without causing a profound reduction in osteoblast activity and bone formation.

In this review, we discuss potential therapies that could be effective in obtaining those effects. Allosteric PTH1R inhibitors like Pitt12 modulate cPTH’s effect on bone metabolism without fully repressing PTH-induced osteoblast activation. Cortical bone loss commonly observed in PHPT was prevented during IL-17A inhibitor treatment without affecting PTH-mediated increases in bone formation. Other agents such as V-ATPase, CTSK, and αvβ3 integrin inhibitors can inhibit the resorptive activity of osteoclasts without affecting their differentiation and osteoclast–osteoblast interactions, allowing the restoration of lost bone mass. While romosozumab, which lowers SOST levels and enhances bone formation, has not been directly studied in PHPT models, it presents a probable therapeutic approach for counteracting the high turnover and resorption levels observed in PHPT.

The limitation of this study is that most of the discussed therapies are either in preclinical stages of development or have not been thoroughly evaluated in the context of PHPT. However, in our opinion, modulators of PTH1R, with their dual ability to exhibit both antiresorptive and anabolic effects on bone, along with IL-17A inhibitors, which help reduce the low-inflammatory state in PHPT, show great promise for future therapeutic strategies. Further research of these agents and others has potential for targeted therapy that could improve the treatment of bone disease in this specific condition, particularly for those for whom surgery is not a viable option.

## Figures and Tables

**Table 1:** Summary of potential and currently recommended therapies in PHPT.

Pharmacological therapies currently recommended in PHPT		
Name of therapy	Mechanism of action	
Bisphosphonates	Inhibition of mevalonate pathway leading to osteoclast dysfunction and apoptosis	
Denosumab	Inhibition of RANKL/RANK pathway	
Cinacalcet	Increasing CaSR sensitivity to extracellular calcium activation, resulting in PTH secretion inhibition; alone it does not improve BMD	
Vitamin D supplementation	Inhibition of parathyroid cells proliferation as well as PTH synthesis and secretion; complex mechanisms of action on calcium metabolism and bone cells leading to increase in BMD values and decrease in BTMs levels	
Therapies in development or needing further study for PHPT		
Name of therapy	Mechanism of action	Development stage
PTH1R inhibition:		
PTH1R orthosteric antagonists (SW106, DS08210767, DS37571084)	Competitive antagonists of PTH1R signaling antagonizing the protein’s active site	Preclinical
Anti-PTH1R antibody (developed by XOMA)	High affinity anti-PTH1R antibody which blocks the activation of PTH1R by PTH	Preclinical
PTH1R negative allosteric modulator—Pit12	Inhibits the binding affinity and catalytic activity of PTH1R by binding to its allosteric regions	Preclinical
IL-17A antibodies	Decrease the level of RANKL-mediated osteoclastogenesis and bone resorption by reducing the IL-17A action on osteocytes and osteoblasts	Clinical use for psoriasis, psoriatic arthritis, and ankylosing spondylitis (secukinumab, ixekizumab, and brodalumab), preclinical studies in PHPT
M-CSF and CSF1R antibodies	Reduce the osteoclast proliferation and differentiation	Preclinical
MCP-1 inhibitor (7ND)	Blockade of RANKL and M-CSF-mediated osteoclast differentiation	Preclinical
αvβ3 integrin inhibitors	Proteins containing RGD-sequence motif or antibodies which inactivate αvβ3 integrin and inhibit osteoclast-mediated bone resorption	Preclinical
V-ATPase inhibition		
SB 242784, FR167356	Osteoclast-specific V-ATPase direct inhibitors	Preclinical
Luteolin	Inhibits the binding between a3 and d3 V-ATPase subunits and reduces osteoclast resorptive activity	Research on luteolin in bone-related diseases is in the preclinical stage, but clinical trials are underway for its use in psychiatric disorders
Bis-enoxacin	Interrupts the interaction between V-ATPase B2 subunit and cell microfilaments. The combination with a bisphosphonate confers additional characteristics typical of this class of drugs	Preclinical
Cathepsin K ectosteric inhibitor	Selectively blocks CTSK’s collagenase activity	Preclinical
Romosuzumab	Anti-sclerostin antibody, leads to increased Wnt/β-catenin signaling and bone formation	Clinical use but in postmenopausal osteoporosis
DKK-1 antibodies	Leads to increased Wnt/β-catenin signaling and bone formation	Preclinical
